# A Novel Statistical Model for Predicting the Efficacy of Vagal Nerve Stimulation in Patients With Epilepsy (Pre-X-Stim) Is Applicable to Different EEG Systems

**DOI:** 10.3389/fnins.2021.635787

**Published:** 2021-05-11

**Authors:** Eva Korit́áková, Irena Doležalová, Jan Chládek, Tereza Jurková, Jan Chrastina, Filip Plešinger, Robert Roman, Martin Pail, Pavel Jurák, Daniel J. Shaw, Milan Brázdil

**Affiliations:** ^1^Institute of Biostatistics and Analyses, Faculty of Medicine, Masaryk University, Brno, Czechia; ^2^Brno Epilepsy Center, Department of Neurology and Neurosurgery, St. Anne’s University Hospital and Medical Faculty of Masaryk University, Brno, Czechia; ^3^Behavioral and Social Neuroscience Research Group, Central European Institute of Technology, Masaryk University, Brno, Czechia; ^4^Institute of Scientific Instruments of the Czech Academy of Sciences, Brno, Czechia

**Keywords:** vagal nerve stimulation, neurostimulation, epilepsy, efficacy prediction, EEG reactivity, epilepsy treatment

## Abstract

**Background:** Identifying patients with intractable epilepsy who would benefit from therapeutic chronic vagal nerve stimulation (VNS) preoperatively remains a major clinical challenge. We have developed a statistical model for predicting VNS efficacy using only routine preimplantation electroencephalogram (EEG) recorded with the TruScan EEG device (Brazdil et al., 2019). It remains to be seen, however, if this model can be applied in different clinical settings.

**Objective:** To validate our model using EEG data acquired with a different recording system.

**Methods:** We identified a validation cohort of eight patients implanted with VNS, whose preimplantation EEG was recorded on the BrainScope device and who underwent the EEG recording according to the protocol. The classifier developed in our earlier work, named Pre-X-Stim, was then employed to classify these patients as predicted responders or non-responders based on the dynamics in EEG power spectra. Predicted and real-world outcomes were compared to establish the applicability of this classifier. In total, two validation experiments were performed using two different validation approaches (single classifier or classifier voting).

**Results:** The classifier achieved 75% accuracy, 67% sensitivity, and 100% specificity. Only two patients, both real-life responders, were classified incorrectly in both validation experiments.

**Conclusion:** We have validated the Pre-X-Stim model on EEGs from a different recording system, which indicates its application under different technical conditions. Our approach, based on preoperative EEG, is easily applied and financially undemanding and presents great potential for real-world clinical use.

## Introduction

Chronic vagal nerve stimulation (VNS) is a well-established non-pharmacological treatment option in patients with drug-resistant epilepsy. VNS offers substantial (≥50%) seizure reduction for approximately 50% of implanted patients (Englot et al., 2011, 2016; Panebianco et al., 2015). Despite the high numbers of implanted patients, however, it is still not possible to identify patients who will benefit from VNS on the basis of preimplantation characteristics alone.

In our previous work (Brazdil et al., 2019), we introduced a statistical model capable of predicting the efficacy of VNS based only on relative power values calculated from standard preoperative interictal scalp electroencephalogram (EEG) recorded during a stimulation protocol with eight conditions (4 × rest, 2 × eyes opening, 1 × photic stimulation, and 1 × hyperventilation). The model was subsequently successfully validated using an independent dataset of 22 patients, achieving 86% accuracy, 83% sensitivity, and 90% specificity. Since our previous work employed only one EEG recording system, in the present study, we aimed to overcome this potential limitation by verifying the applicability of our model to EEG data recorded with a different system.

## Materials and Methods

### Study Design and Electroencephalogram Data

In our previous study (Brazdil et al., 2019), the statistical model, named “Pre-X-Stim,” was developed using 19-channel EEG data acquired from 60 patients (35 responders and 25 non-responders) using the TruScan EEG device (Deymed Diagnostic, Czechia) with electrodes placed according to the 10–20 system, a sampling frequency of 128 Hz, and the reference electrode AFz placed in the middle of Fz, Fp1, and Fp2. These previous data served as the training cohort here.

In the present study, the validation cohort comprised patients with preimplantation 19-channel EEG recorded using the BrainScope device (M&I, Czechia) with the 10–20 electrode system, a sampling frequency of 1,000 Hz, and the reference representing an average of the vertex electrodes Fz, Cz, and Pz. We estimated outcomes for patients comprising this cohort (i.e., responders or non-responders of VNS) using the Pre-X-Stim model. To evaluate the applicability of the model, these predicted outcomes were compared with real-life outcomes obtained by analyzing patients’ seizure diaries using the McHugh classification (McHugh et al., 2007).

All patients from both cohorts were recruited in St. Anne’s University Hospital, Brno, Czechia. The hospital’s institutional review board approved the study, and all patients provided written informed consent prior to the experimental procedure.

### Signal Processing

Since the BrainScope system uses a different reference, data were re-referenced to an average from Fz, Fp1, and Fp2 electrodes (Supplementary Material). Afterwards, data were filtered and resampled to 128 Hz to match EEG signals from the training cohort. Subsequently, EEG signals from the validation cohort were processed in the same way as EEGs from the training cohort. Specifically, EEG signals were transformed into power envelopes in four standard frequency bands using the fast Fourier and Hilbert transforms: theta (4–7.5 Hz), alpha (8–12 Hz), beta (14–30 Hz), and gamma (31–45 Hz). Band power estimates were segmented into eight time intervals corresponding to the eight conditions in the stimulation protocol:

1 – Rest #1 (2 min);

2 – Eyes opening/closing (10 s);

3 – Rest #2 (immediately after eye closure; 10 s);

4 – Photic stimulation [2.5 min; stimulation frequencies: 5 Hz (10 s) – 10 Hz (10 s) – 15 Hz (10s) – 20 Hz (10 s) – 25 Hz (10 s) – 30 Hz (10 s) – 25 Hz (5 s) – 20 Hz (5 s) – 15 Hz (5 s) – 10 Hz (5 s) – 5 Hz (5 s); light intensity at least 0.7 Joule];

5 – Hyperventilation (4 min – 2 min by nose and 2 min by mouth);

6 – Eyes opening/closing (10 s);

7 – Rest #3 (immediately after eye closure; 10 s);

8 – Rest #4 (2 min).

Finally, their mean values were normalized to a baseline representing the first rest interval. Further details regarding the EEG signal processing are specified in Brazdil et al. (2019).

### Statistical Analysis

Non-parametric Mann–Whitney and Fisher’s exact tests were applied to compare clinical data from the two cohorts.

The validation dataset was reduced by selecting eight maximally discriminative electrode groups within anatomical areas defined from the training dataset based on stepwise logistic regression performed in leave-one-out manner (Brazdil et al., 2019); specifically, the right frontal region (Fp2, F4, and Fz) in the Eyes opening/closing condition in the beta frequency band as well as in the Rest #2 condition in the beta and theta frequency bands, the right posterior quadrant (P4, Pz, T6, and O2) in the Eyes opening/closing condition in the alpha frequency band, the left posterior quadrant (P3, Pz, T5, and O1) in the Hyperventilation condition in the alpha and beta frequency bands, the central region (C3, Cz, and C4) in the Hyperventilation condition in the gamma frequency band, and the right anterotemporal region (F8 and T4) in the Rest #4 condition in the theta frequency band.

**FIGURE 1:**
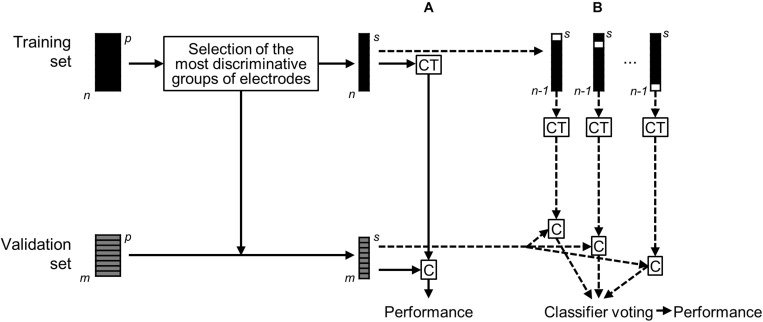
Schematics depicting validation of the statistical model. Only the eight most discriminative groups of electrodes defined from the training set were selected. Then, classifier training (CT) based on the reduced training set of *n* = 60 patients and classification (C) of the reduced validation set of *m* = 8 patients was performed in two approaches: **(A)** a single classifier trained on all *n* subjects from the training set; **(B)** voting of *n* classifiers trained on *n*–1 subsets of the training set.

The Pre-X-Stim model was then validated via two approaches (Figure 1). In the first validation approach, subjects from the validation cohort were classified using linear discriminant analysis as predicted responders or non-responders based on a single classifier, which was trained on all *n* subjects from the training set. In the second approach, the classification of subjects from the validation cohort was performed using majority voting of *n* classifiers trained on *n*−1 subsets of the training set. The predicted outcomes were compared with real-life outcomes to determine the accuracy, sensitivity, and specificity of the model.

## Results and Discussion

We identified eight patients (six real-life responders and two non-responders) treated with VNS, in whom EEG was recorded with the BrainScope system according to the published protocol. When comparing their clinical characteristics to patients in the training set, we found statistical differences in the age of epilepsy onset, duration of VNS, and treatment with levetiracetam and valproid acid (Table 1). Specifically, the eight patients from the validation set had significantly higher age at epilepsy onset (*p* = 0.024), had slightly shorter duration of VNS (*p* = 0.001), and were more often treated with valproic acid, and none of them was treated with levetiracetam (*p* = 0.034 and *p* = 0.001, respectively). The individual characteristics of the eight patients are given in Table 2.

**TABLE 1:** Demographic and treatment data for the training and validation datasets.

		**Training set (*n* = 60)**	**Validation set (*n* = 8)**	**p-Value**
Type of epilepsy, *n* (%)	TLE	14 (23)	3 (38)	0.605
	Extra-TLE	43 (72)	5 (63)	
	IGE	3 (5)	0 (0)	
Gender, *n* (%)	Females	34 (57)	3 (38)	0.454
	Males	26 (43)	5 (63)	
Age (years) at epilepsy onset (median, min–max)		9 (1–51)	16 (7–60)	0.024
Age (years) at VNS implantation (median, min–max)		33 (15–65)	34 (22–71)	0.947
Duration (years) of epilepsy before VNS implantation (median, min–max)		22 (4–60)	16 (2–30)	0.116
Duration (years) of VNS treatment (median, min–max)		6 (3–11)	4 (3–5)	0.001
Treatment at the time of VNS implantation, *n* (%)	BRV	2 (3)	0 (0)	1.000
	CBZ	32 (53)	3 (38)	0.471
	CLB	1 (2)	0 (0)	1.000
	CLZ	13 (22)	3 (38)	0.380
	ESL	3 (5)	1 (13)	0.401
	GBP	1 (2)	1 (13)	0.223
	LCM	6 (10)	2 (25)	0.236
	LEV	36 (60)	0 (0)	0.001
	LTG	27 (45)	3 (38)	1.000
	PGB	5 (8)	1 (13)	0.543
	PHE	1 (2)	0 (0)	1.000
	PHT	4 (7)	1 (13)	0.476
	PRM	3 (5)	0 (0)	1.000
	TPM	13 (22)	1 (13)	1.000
	VPA	14 (23)	5 (63)	0.034
	ZNS	8 (13)	3 (38)	0.113
Number of AEDs used at the time of VNS implantation, *n* (%)	1	4 (7)	0 (0)	0.468
	2	17 (28)	3 (38)	
	3	26 (43)	3 (38)	
	4	12 (20)	1 (13)	
	5	1 (2)	1 (13)	

*AEDs, antiepileptic drugs; BRV, brivaracetam; CBZ, carbamazepine; CLB, clobazam; CLZ, clonazepam; ESL, eslicarbazepine; Extra-TLE, extratemporal lobe epilepsy; GBP, gabapentin; IGE, idiopathic generalized epilepsy; LCM, lacosamide; LEV, levetiracetam; LTG, lamotrigine; *n*, number of patients; PGB, pregabalin; PHE, phenobarbital; PHT, phenytoin; PRM, primidone; TLE, temporal lobe epilepsy; TPM, topiramate; VNS, vagal nerve stimulation; VPA, valproic acid; ZNS, zonisamide.*

**TABLE 2:** Characteristics of individual patients from the validation set.

**ID**	**Gender**	**Type of epilepsy**	**Seizure type**	**Seizure frequency before implantation **/ month****	**MRI**	**EEG**	**Age (years) at epilepsy onset**	**Age (years) at VNS implantation**	**Duration (years) of epilepsy before VNS implantation**	**Duration (years) of VNS treatment**	**Treatment at the time of VNS implantation**	**Responder to VNS**
1*	M	Extra-TLE	FIAS, FBTCS	3	Bilateral posttraumatic changes	BiF	20	22	2	5	VPA, TPM	Yes
2	M	Extra-TLE	FAS, FIAS, FBTCS	30–60	Bilateral schizencephaly with polymicrogyria	BiF	7	22	15	3	ZNS, CBZ, LCM, CLZ	Yes
3	M	Extra-TLE	FIAS, FBTCS	60	Normal	Independent over LF and LT	23	39	16	4	LCM, GBP	Yes
4*	F	Extra-TLE	FIAS, FBTCS	>50	Normal	Generalized slight preponderance over LF	8	28	20	3	LTG, VPA	Yes
5	F	TLE	FAS, FIAS	30	Widely distributed gliosis in LT and LF lobe	LF	30	53	23	3	ZNS, CLZ, VPA	Yes
6	F	TLE	FIAS, FBTCS	5	Normal	RT	60	71	11	4	CBZ, ZNS, PGB	Yes
7	M	Extra-TLE	FIAS	90	Large resection^#^ of RF lobe, no other changes	RF	11	25	14	4	ESL, LTG VPA, PHT, CLZ	No
8	M	TLE	FIAS, FBTCS	15	Resection^#^ of LT lobe, posttraumatic changes in RF, arachnoidal cyst in LT	Multifocal over LH	12	42	30	4	CBZ, VPA, LTG	No

*incorrectly classified patient. ^#^surgery for drug-resistant epilepsy. BiF, bifrontal; BRV, brivaracetam; CBZ, carbamazepine; CLB, clobazam; CLZ, clonazepam; ESL, eslicarbazepine; Extra-TLE, extratemporal lobe epilepsy; FAS, focal aware seizure; FBTCS, focal to bilateral tonic–clonic seizure; FIAS, focal impaired awareness seizure; GBP, gabapentin; LCM, lacosamide; LEV, levetiracetam; LF, left frontal; LH, left hemisphere; LT, left temporal; LTG, lamotrigine; PGB, pregabalin; PHE, phenobarbital; PHT, phenytoin; PRM, primidone; RF, right frontal; RT, right temporal; TLE, temporal lobe epilepsy; TPM, topiramate; VNS, vagal nerve stimulation; VPA, valproic acid; ZNS, zonisamide.

The classification efficiency was the same for the two validation experiments; we reached 75% accuracy, 67% sensitivity, and 100% specificity. The accuracy is comparable to the accuracy of 86% obtained using the validation set of 22 patients in our previous study (two-sample binomial test, *p* = 0.478). In this study, both validation experiments failed in two patients only. A first incorrectly classified patient is a man treated with focal extratemporal epilepsy, who is after 5 years of VNS therapy completely seizure-free. The second incorrectly classified case is disputable. It is a female patient treated with frontal lobe epilepsy with focal cortical dysplasia who refused brain surgery for personal reasons. Before VNS implantation, she mainly complained about bilateral tonic–clonic seizures with high frequency (two seizures per week). She also admitted focal seizures with impaired awareness with unknown frequency. After VNS implantation, the bilateral tonic–clonic seizures completely disappeared, and the frequency of focal seizures with impaired awareness decreased. We classified this patient as a real-life responder because of the improvement of quality of life caused by VNS. Still, the exact percentage reduction in the numbers of seizures is not available.

The most prominent advantage of our approach is the high accessibility of EEG with minimal burden placed upon the patient while maintaining the efficacy comparable to that of other studies. Our approach is based on the application of two routinely used activation methods, namely, photic stimulation and hyperventilation. Photic stimulation causes changes of cerebral blood flow over occipital areas, as proven by functional magnetic resonance imaging (fMRI) and positron emission tomography (Frahm et al., 1992; Hedera et al., 1998). It is known that hyperventilation activates both slowing of EEG and interictal epileptiform abnormality. However, the exact mechanism of its action is not fully understood. There are debates, whether these changes, i.e., slowing and presence of interictal epileptiform abnormality, are dependent on or independent of each other and are conditioned by the brain’s hypoxia or hypocapnia (Kennealy et al., 1986; Guaranha et al., 2005).

Prediction of VNS response using diffusion tensor imaging (DTI) data has achieved 83.3% accuracy, 85.7% sensitivity, and 75.0% specificity in a validation set comprising 18 pediatric patients (Mithani et al., 2019). Another study focused on thalamocortical connectivity in resting-state fMRI shows 88% accuracy, 100% sensitivity, and 0% specificity in eight external patients (Ibrahim et al., 2017). A third study utilizing magnetoencephalography (MEG) achieved an accuracy of 85, 80, and 95% in the classification of 9 non-responders, 14 responders, and 14 controls, respectively (Babajani-Feremi et al., 2018). Despite its interesting results, the last study is limited due to the lack of an external validation set.

The main limitation of our study is the small number of patients in the validation set and their uneven distribution between responders and non-responders. Regardless of these shortcomings, however, our results are a promising cornerstone for our planned prospective multicenter study. Besides, power analysis showed an estimation of power equal to 47.9% for a margin of 70% in a non-inferiority investigation demonstrating that the 75% accuracy obtained using EEG data of 8 patients acquired using BrainScope device is not worse than the 86% accuracy obtained using EEGs of 22 patients acquired using TruScan EEG device. The achieved power is well above the estimated median statistical power of studies in the neuroscience fields ranging between 8% and 31% (Button et al., 2013).

## Conclusion

We provided evidence that our Pre-X-Stim model for the prediction of VNS response is not dependent on the type of EEG recording system, which indicates its universal applicability. The main advantage of our approach is the high accessibility of standard scalp-recorded EEG relative to other methods, such as the DTI, fMRI, or MEG data employed previously. This enhances the potential of our Pre-X-Stim model to become a tool used widely in real-world clinical practice.

## Data Availability Statement

The raw data supporting the conclusions of this article will be made available by the authors, without undue reservation.

## Ethics Statement

The studies involving human participants were reviewed and approved by the Institutional Review Board of St. Anne’s University Hospital, Brno, Czechia. Written informed consent to participate in this study was provided by the participants’ legal guardian/next of kin.

## Author Contributions

EK contributed to the preparation of the manuscript, statistical analysis, and interpretation of results. ID and MP contributed to the preparation of study design, data collection, and interpretation of results. JChl and FP performed the mathematical analysis. TJ performed the statistical analysis. RR, PJ, JChr, DS, and MB contributed to the identification of research topic, preparation of study design, and interpretation of results. All authors critically revised the manuscript and approved the final version.

## Conflict of Interest

The authors declare that the research was conducted in the absence of any commercial or financial relationships that could be construed as a potential conflict of interest.

## References

[B1] Babajani-Feremi A. Noorizadeh N. Mudigoudar B. Wheless J. W. (2018). Predicting seizure outcome of vagus nerve stimulation using MEG-based network topology. *NeuroImage Clin.* 19 990–999. 10.1016/j.nicl.2018.06.017 30003036 PMC6039837

[B2] Brazdil M. Dolezalova I. Koritakova E. Chladek J. Roman R. Pail M. (2019). EEG reactivity predicts individual efficacy of vagal nerve stimulation in intractable epileptics. *Front. Neurol.* 10:392. 10.3389/fneur.2019.00392 31118916 PMC6507513

[B3] Button K. S. Ioannidis J. P. A. Mokrysz C. Nosek B. A. Flint J. Robinson E. S. J. (2013). Power failure: why small sample size undermines the reliability of neuroscience. *Nat Rev Neurosci* 14 365–376. 10.1038/nrn3475 23571845

[B4] Englot D. J. Chang E. F. Auguste K. I. (2011). Vagus nerve stimulation for epilepsy: a meta-analysis of efficacy and predictors of response. *J. Neurosurg.* 115 1248–1255. 10.3171/2011.7.JNS11977 21838505

[B5] Englot D. J. Rolston J. D. Wright C. W. Hassnain K. H. Chang E. F. (2016). Rates and predictors of seizure freedom with vagus nerve stimulation for intractable epilepsy. *Neurosurgery* 79 345–353. 10.1227/NEU.0000000000001165 26645965 PMC4884552

[B6] Frahm J. Bruhn H. Merboldt K.-D. Hanicke W. (1992). Dynamic MR imaging of human brain oxygenation during rest and photic stimulation. *J. Magn. Reson. Imaging* 2 501–505. 10.1002/jmri.1880020505 1392241

[B7] Guaranha M. S. B. Garzon E. Buchpiguel C. A. Tazima S. Yacubian E. M. T. Sakamoto A. C. (2005). Hyperventilation revisited: physiological effects and efficacy on focal seizure activation in the era of video-EEG monitoring. *Epilepsia* 46 69–75. 10.1111/j.0013-9580.2005.11104.x 15660770

[B8] Hedera P. Wu D. Collins S. Lewin J. S. Miller D. Lerner A. J. (1998). Sex and electroencephalographic synchronization after photic stimulation predict signal changes in the visual cortex on functional MR images. *Am. J. Neuroradiol.* 19 853–857. 9613499 PMC8337582

[B9] Ibrahim G. M. Sharma P. Hyslop A. Guillen M. R. Morgan B. R. Wong S. (2017). Presurgical thalamocortical connectivity is associated with response to vagus nerve stimulation in children with intractable epilepsy. *NeuroImage Clin.* 16 634–642. 10.1016/j.nicl.2017.09.015 28971013 PMC5619991

[B10] Kennealy J. A. Penovich P. E. Moore Nease S. E. (1986). EEG and spectral analysis in acute hyperventilation. *Electroencephalogr. Clin. Neuropsychiol.* 63 98–106. 10.1016/0013-4694(86)90002-7 2417822

[B11] McHugh J. C. Singh H. W. Phillips J. Murphy K. Doherty C. P. Delanty N. (2007). Outcome measurement after vagal nerve stimulation therapy: proposal of a new classification. *Epilepsia* 48 375–378. 10.1111/j.1528-1167.2006.00931.x 17295633

[B12] Mithani K. Mikhail M. Morgan B. R. Wong S. Weil A. G. Deschenes S. (2019). Connectomic profiling identifies responders to vagus nerve stimulation. *Ann. Neurol.* 86 743–753. 10.1002/ana.25574 31393626

[B13] Panebianco M. Rigby A. Weston J. Marson A. G. (2015). Vagus nerve stimulation for partial seizures. *Cochrane Database Syst. Rev.* 2015:CD002896. 10.1002/14651858.CD002896.pub2 PMC7138043 25835947

