# Supplementary material for: A Novel Statistical Model for Predicting the Efficacy of Vagal Nerve Stimulation in Patients With Epilepsy (Pre-X-Stim) Is Applicable to Different EEG Systems
Source: Front Neurosci. 2021 May 11;15:635787. doi: 10.3389/fnins.2021.635787 (PMC8144700; doi:10.3389/fnins.2021.635787)
Supplement: Supplementary file 1 [file Image_1.pdf]

Supplementary Material

Detailed description of re-referencing in EEG data

Reference electrodes in both recording systems are shown in Supplementary Figure 1. The TruScan EEG (Deymed Diagnostic, Czechia) recording device uses online reference electrode AFz placed in the middle of Fp1, Fp2, and Fz electrode positons (Supplementary Figure 1A). In the BrainScope (M&I, Czechia) recording system, a reference corresponds to an average of the vertex electrodes Fz, Cz, and Pz (Supplementary Figure 1B).

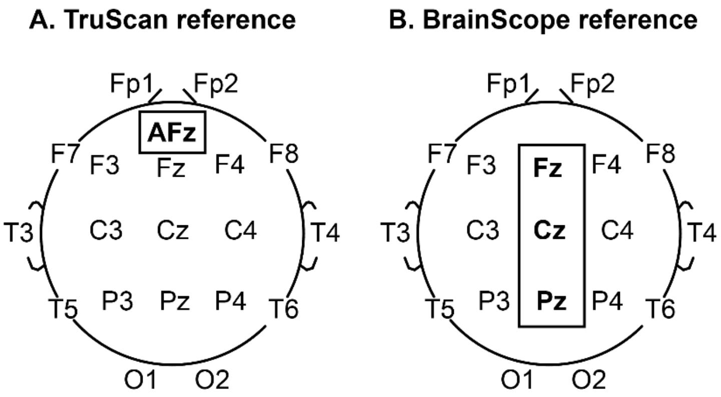

Supplementary Figure 1. Electrode placement in the international 10-20 system for (A) training and (B) validation recordings. Reference electrodes are highlighted.

Each recorded EEG channel in the BrainScope system was offline re-referenced to make the potentials comparable to the TruScan EEG system. Re-referencing is a linear transformation performed by subtracting a new reference channel from all the originally recorded channels. Any recorded channel or an average of two or more channels can serve as a new reference channel (1-3). Specifically, re-referencing of the BrainScope EEG data can be described in the following steps:

1) Calculation of a new reference (ref_{new}), which corresponds to the position of the electrode AFz in the TruScan EEG device, as an average over surrounding sensors:

$$ref_{new} = {}^{1}/_{3}\ (F_z + Fp_1 + Fp_2).$$

As electrode pairs represent the potential difference between two electrodes, this new reference by itself is related to the original reference of the BrainScope system, i.e. $[ref_{new} - ref_{old}]$, where $ref_{old} = {}^{1}/_{3}\ (F_z + C_z + P_z)$.

2) Relating the BrainScope EEG data to the new reference. Signal of the new reference is extracted from each EEG channel which has to be re-referenced:

$$[channel - ref_{old}] - [ref_{new} - ref_{old}] \Rightarrow [channel - ref_{new}],$$

where *channel* refers to any of EEG channels stored from the BrainScope system.

References:

1. Osselton JW. Acquisition of EEG data by bipolar unipolar and average reference methods: a theoretical comparison. *Electroencephalogr Clin Neurophysiol* (1965) 19(5):527–8. doi: 10.1016/0013-4694(65)90195-1
2. Luck SJ. An introduction to the event-related potential technique. Cambridge: MIT press (2014).
3. Leuchs L. Choosing your reference – and why it matters. *Brain Products* (2019) 1:1–4.
